# Using Immersive Virtual Reality Distraction to Reduce Fear and Anxiety before Surgery

**DOI:** 10.3390/healthcare11192697

**Published:** 2023-10-09

**Authors:** Araceli Flores, Hunter G. Hoffman, Maria Vicenta Navarro-Haro, Azucena Garcia-Palacios, Barbara Atzori, Sylvie Le May, Wadee Alhalabi, Mariana Sampaio, Miles R. Fontenot, Keira P. Mason

**Affiliations:** 1 Ben Taub Hospital Psychiatry and Behavioral Sciences, Baylor College of Medicine, Houston, TX 77030, USA; 2 William Beaumont Army Medical Center, Fort Bliss, TX 79918, USA; 3 El Paso VA Health Care System, Veterans Health Administration, United States Department of Veterans Affairs, El Paso, TX 79930, USA; 4 Department of Mechanical Engineering, University of Washington, Seattle, WA 98195, USA; 5 Department of Psychology and Sociology, University of Zaragoza, 44003 Teruel, Spain; 6 Instituto de Investigación Sanitaria de Aragón (IISA), 50009 Zaragoza, Spain; 7 Department of Basic Psychology, Clinic and Psychobiology, Jaume I University, 12006 Castelló de la Plana, Spain; azucena@uji.es; 8 Department of Health Sciences, University of Florence, 50121 Florence, Italy; psicob.atzori@gmail.com; 9 Centre de Recherche du CHU Sainte-Justine, Université de Montréal, Montreal, QC H3T 1J4, Canada; sylvie.lemay@umontreal.ca; 10 Centre de Recherche de l’Institut, Universitaire en Santé Mentale de Montréal (CRIUSMM), Montreal, QC H1N 3M5, Canada; 11 Faculty of Nursing, University of Montreal, Montreal, QC H1N 3M5, Canada; 12 Department of Computer Science, Faculty of Computing and Information Technology, King Abdulaziz University, Jeddah 21589, Saudi Arabia; 13 Department of Computer Science, Dar Alhekma University, Jeddah 21589, Saudi Arabia; 14 Department of Social Work, Catholic University of Portugal, 1649-023 Lisboa, Portugal; mari@mindovermatterinstitute.com; 15 Department of Psychology, University of Coimbra, 1649-023 Lisboa, Portugal; 16 Department of Anesthesiology & Pain Medicine, School of Medicine, University of Washington, Seattle, WA 98195, USA; 17 Department of Anesthesiology, Critical Care and Pain Medicine, Harvard Medical School, Boston Children’s Hospital, Boston, MA 02115, USA; keira.mason@childrens.harvard.edu

**Keywords:** sedation, analgesia, distraction, nonpharmacologic analgesic techniques, opioid, pain, virtual reality, digital therapeutics, mHealth, healthcare

## Abstract

Presurgical anxiety is very common and is often treated with sedatives. Minimizing or avoiding sedation reduces the risk of sedation-related adverse events. Reducing sedation can increase early cognitive recovery and reduce time to discharge after surgery. The current case study is the first to explore the use of interactive eye-tracked VR as a nonpharmacologic anxiolytic customized for physically immobilized presurgery patients. Method: A 44-year-old female patient presenting for gallbladder surgery participated. Using a within-subject repeated measures design (treatment order randomized), the participant received no VR during one portion of her preoperative wait and interactive eye-tracked virtual reality during an equivalent portion of time in the presurgery room. After each condition (no VR vs. VR), the participant provided subjective 0–10 ratings and state–trait short form Y anxiety measures of the amount of anxiety and fear she experienced during that condition. Results: As predicted, compared to treatment as usual (no VR), the patient reported having 67% lower presurgical anxiety during VR. She also experienced “strong fear” (8 out of 10) during no VR vs. “no fear” (0 out of 10) during VR. She reported a strong sense of presence during VR and zero nausea. She liked VR, she had fun during VR, and she recommended VR to future patients during pre-op. Interactive VR distraction with eye tracking was an effective nonpharmacologic technique for reducing anticipatory fear and anxiety prior to surgery. The results add to existing evidence that supports the use of VR in perioperative settings. VR technology has recently become affordable and more user friendly, increasing the potential for widespread dissemination into medical practice. Although case studies are scientifically inconclusive by nature, they help identify new directions for future larger, carefully controlled studies. VR sedation is a promising non-drug fear and anxiety management technique meriting further investigation.

## 1. Introduction

Preoperative anxiety is very common, experienced by an estimated 30–50% of patients undergoing surgery [1]. “Severe anxiety can cause unpleasant symptoms and stress. Typical symptoms include a pounding heart, a racing heart (fast pulse), irregular heartbeat, nausea, a nervous stomach, shortness of breath and sleep problems” [2]. Furthermore, patients who have heart problems may feel more heart pain as a result of the anxiety. Although some anxiety before surgery is normal, patients with severe anxiety are especially likely to be administered anxiolytic sedative medications (e.g., benzodiazepines) to help calm them and reduce their anxiety as they are waiting and being prepared for surgery. For pediatric patients, “High levels of perioperative anxiety have been associated with a multitude of negative outcomes, including the prolonged induction of anaesthesia, increased incidence of postoperative delirium, new onset negative postoperative behaviour changes related to anxiety, increased postoperative pain, and increased use of analgesics” Agbayani et al., 2020, (p. 424) [3]; see also [4,5]. Although reducing anxiety is important, pharmacologic sedation has side effects [6]. Whenever possible, avoidance of sedation or “sedation sparing” is preferred in order to improve efficiency, reduce the risk of sedation-related adverse events [7], eliminate the often out-of-pocket sedation-related expenses to patients, allow for discharge home without an escort, and improve patient satisfaction. Periprocedure anxiety (e.g., patients in the preoperative holding area waiting room before surgery) is frequently the impetus for requiring the administration of anxiolytic medications and sedatives. 

One approach is to use augmented reality and/or immersive virtual reality to help educate patients about what to expect on their day of surgery and to walk them through the surgery space. Explaining procedures to patients in advance can help reduce anticipatory anxiety [8,9,10]. 

VR distraction (used in the present study) is another approach. Nonpharmacologic techniques using distraction are being applied during a growing number of medical procedures to provide anxiolysis with the goal of reducing or eliminating the need for sedative administration [11,12]. Immersive virtual reality (VR) is emerging as a sophisticated and effective non-drug technique for anxiety and pain reduction [13,14,15,16,17,18,19,20,21,22,23,24,25,26,27,28,29,30], e.g., during venipuncture [29,30] and even during surgery [31]. fMRI studies show that VR reduces pain-related brain activity, and the amount of pain reduction during VR is comparable to a moderate dose of opioids in analgesic effectiveness [32,33,34]. Although the mechanism of how VR reduces fear and anxiety is not well understood, there is growing evidence that VR floods the brain with information, reducing the amount of attentional resources available [35,36]. We speculate that this reduction in attentional resources during VR may help reduce pathological thought processes associated with the amplification of fear and anxiety, such as rumination and catastrophizing [4,37,38]. Put simply, during VR, patients may spend less time worrying about what is going to happen during surgery and less time thinking about whether the surgery will be successful, because their attention is focused on interacting with the virtual world in the present moment; see also [39].

Regional anesthesia is often safer than general anesthesia; e.g., it reduces possible oversedation side effects. Patients typically remain conscious during surgery, making regional anesthesia an excellent procedure for adding VR distraction. A recent study compared the effects of premedication with virtual reality distraction vs. midazolam in patients undergoing surgery under combined spinal epidural (CSE) regional anesthesia [31]. The results showed a significantly lower anxiety score in the VR group than in the control group during surgery. However, presurgery was a different story. The patients were highly anxious during presurgery, even when using passive VR (noninteractive 360 meditation videos). A more immersive VR system strong enough to reduce intense presurgery anxiety was tested in the current study.

Laboratory studies have shown that, compared to passive VR, interacting with virtual objects in the virtual world made VR significantly more effective at reducing pain during a brief thermal stimulus, and interactivity also significantly increased fun (a surrogate measure of positive emotional affect). In most virtual worlds, interacting with the virtual world requires physically moving the hands and arms around in the real world (e.g., reaching out your “position tracked” real hand in the real world to pick up a virtual object in VR). Patients in the pre-op room with IVs in their arms should keep their arms still and should not use traditional hand-tracked interactivity, but limiting interactivity reduces VR analgesia.

The current study takes an innovative look at using interactive eye-tracked VR to alleviate presurgical anxiety. The current study explores for the first time whether enabling a physically immobilized patient in the pre-op room to interact with virtual objects in virtual reality (VR) via “hands free” eye-tracking technology integrated into the VR helmet could help actively engage the subject in the immersive virtual world SnowCanyon. SnowCanyon is a research VR system designed in collaboration with HH, created and owned by BigEnvironments.com. SnowCanyon is customized to distract patients who need to remain physically still (e.g., in their gurney with IVs in their arms during pre-op). This case study measured, for the first time, the ability and feasibility of using immersive virtual reality to reduce preoperative anxiety and to reduce fear in a female patient presenting for gallbladder surgery. If effective, in the future, VR may improve outcomes and potentially reduce the need for preoperative sedation.

## 2. Materials and Methods

This study was conducted in compliance with the Declaration of the World Medical Association (https://www.wma.net/what-we-do/medical-ethics/declaration-of-helsinki/ accessed on 4 October 2023). After establishing that the patient understood the plan, written consent was obtained using a protocol approved by the IRB for Baylor College of Medicine and the affiliated hospitals’ approval (Protocol Number: H-41124, PI Araceli Flores, Ph.D.). Inclusion and exclusion criteria were established for this study. The inclusion criteria were (1) patients who were scheduled to have surgery on the Acute Care Surgery Service (ACS) at Ben Taub Hospital in Houston, TX, with diagnoses of undergoing cholecystectomy; skin, soft tissue, and bony surgical debridement; excision; and/or amputation; (2) patients who were in the adult patient population (ages 18 to 65); (3) patients who were physically able to participate in the VR intervention, not critically ill or medically compromised (e.g., no tracheotomy, infections, or head wounds that preclude using a head mounted VR system); and (4) patients who were English speakers. The exclusion criteria were (1) patients who were not capable of answering questions and/or not able to fill out the study measures; (2) patients who were lacking the intellectual capacity to give informed consent; (3) patients who were demonstrating delirium or psychosis; (4) patients who had extreme susceptibility to motion sickness; and (5) patients who had seizure history or medical issues that precluded the use of virtual reality. The participant was a 44-year-old bilingual Hispanic female with no significant previous medical history who presented with 3 days of right upper quadrant pain associated with decreased oral intake, nausea, and chills. Ultrasound was negative for stones and negative for ductal dilatation. A computerized tomography (CT) scan showed a mildly thickened gallbladder wall. A hepatobiliary iminodiacetic acid (HIDA) scan tested positive for acute cholecystitis. Blood tests indicated white blood cells 15. Liver enzymes were within normal limits. The patient was scheduled to receive a laparoscopic cholecystectomy, also known as minimally invasive cholecystectomy (surgical removal of the gallbladder). If left untreated, an infected gallbladder can lead to life-threatening infections that can spread to other parts of the body. 

Equipment. This study used an HTC VIVE VR helmet with scientific-grade Sensory Motoric Instruments eye-tracking technology integrated inside the helmet. Several small light sources were located around the lens of the VR goggles, and the patterns of light reflections on the patient’s eyes were used to monitor where the patient was looking in the virtual world. The patient could interact with the virtual world using eye movements/eye fixations to select objects and throw snowballs in VR (e.g., at snowmen). The VR system received images from an MSI gaming laptop connected to the SMI HTC VIVE VR helmet with a wide field of view of 110° from the HTC, with 1200 × 1080 pixels per eye at 90 Hz. A wide field of view and high resolution help increase the immersiveness of the VR system, ideally with low power consumption to avoid overheating [40].

**Figure 1:**
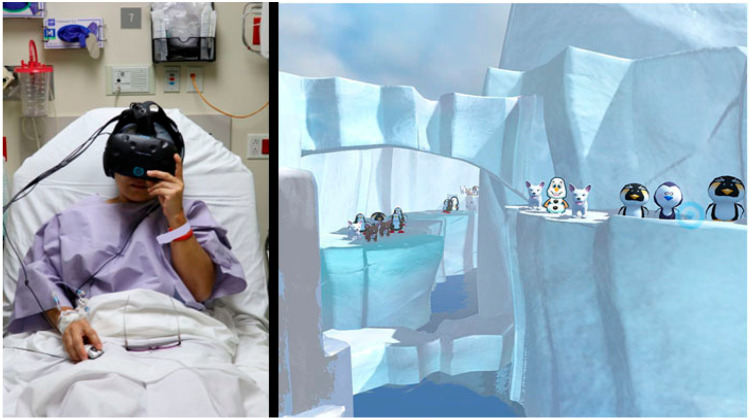
(**left**): The patient using eye tracking and a mouse to interact with an immersive virtual world named SnowCanyon (shown on the **right**) as she waited in the pre-op room to have surgery for gallbladder removal (left photo and copyright Hunter Hoffman, vrpain.com, right image bigenvironments.com, copyright Hunter Hoffman, www.vrpain.com). Both images used with permission.

While in the preoperative holding area (i.e., the pre-op room) and while being prepared to go into the operating room for surgery, the patient spent 10 min with no VR vs. 10 min in SnowCanyon VR distraction (the treatment order was randomized). To collect data and reduce carryover effects, there was a 3 min “washout” period after the first 10 min treatment condition. During the washout period, the patient filled out brief paper and pencil questionnaires. In the SnowCanyon virtual reality distraction, the patient floated slowly through a 3D canyon with a virtual river at the bottom of the canyon (see Figure 1). As described in more technical detail by [41], using the SMI eye-tracking technology embedded in the VR goggles, the patient simply looked at (fixated upon) virtual objects to aim, and left clicked the computer mouse to interact with the virtual world by throwing snowballs at objects in the virtual world (e.g., snowmen and penguins). The snowmen and penguins reacted with animated responses and toyish squeaks when hit by a virtual snowball. Using emotion-driven design [42], SnowCanyon aims to elicit predetermined positive emotions (fun, or positive affect) and to reduce negative emotions (e.g., fear). SnowCanyon is also specifically designed to minimize simulator sickness. 

Measures. The visual analog scale measures (shown in the Supplementary Materials) were administered after each treatment condition. After each session, the participant rated her fear (0 = no fear; 10 = extremely high fear) and anxiety/nervousness (0 = not anxious/nervous at all; 10 = extremely anxious/nervous) and answered several questions about her VR experience using Graphic Rating Scales (GRSs), a rating scale that has previously been shown to be valid [43,44]. After VR, using GRS questions, she rated how much fun she had during VR, how real she found the virtual objects, how sick to her stomach she felt (0 = not sick at all; 10 = throw up), how much she liked VR, whether she would recommend VR to other patients waiting for surgery, and her illusion of “being there” in the computer-generated world (presence). In addition to the single GRS anxiety rating, anxiety was also measured on a scale from 1 to 4, using the 10-item Short STAI-Y anxiety scales (10 state items and 10 trait items, for a total of 20 items after each 10 min session). This scale has high validity and test–retest reliability [45,46]; see also [47,48]. 

## 3. Results

While waiting in the pre-op room, on a single GRS scale rating from 0 to 10 (see the Supplementary Materials for full questions and responses), the patient reported being “moderately anxious” during no VR (6 out of 10 on the GRS measure) vs. only “mildly anxious” during VR (2 out of 10). On the 0–40 state–trait anxiety measure, short form Y, she rated her state anxiety as 20 (moderate anxiety) during no VR, and this dropped to 10 (mild anxiety) during VR. Her trait anxiety was 15. Similarly, the patient reported having “strong fear” (rated 8 out of 10) in the pre-op room during no VR vs. zero fear during VR in the pre-op room. On the GRS ratings, on a scale from 0 to 10, she reported that VR in the pre-op room was extremely fun (rated 10 out of 10), that it was pretty real (rated 8 out of 10), that she had no nausea (rated 0), that she liked VR (rated 8 out of 10), that she would recommend VR to other patients to use during VR during pre-op (rated 9 out of 10), and that she had a strong illusion of “being there” as if the virtual world was a place she visited (rated 8 out of 10). 

## 4. Discussion

The current study introduced a nonpharmacological approach, supported by data showing reduced fear and anxiety levels. Although one previous study using noninteractive 360 videos did not reduce pre-op anxiety [31], in the current study, interacting with a computer-generated immersive virtual reality world successfully reduced the fear and anxiety of a female patient while she waited to go into the operating room to have gallbladder surgery. The patient reported having a strong illusion of presence in VR and the illusion of “being there” in the 3D computer-generated world. She gave high ratings regarding how much fun she had in VR and how real the objects in virtual reality seemed. Regarding potential adverse events of VR in this context, the patient reported zero nausea from using VR. She liked VR, and she recommended VR during pre-op to other patients. Overall, immersive VR improved the patient’s preoperative experience, and she recommended VR to future patients. The results add to existing evidence that supports the use of VR in a perioperative setting. For example, one recent meta-analysis found that virtual reality significantly reduced preoperative anxiety in children and significantly increased children’s compliance with anesthesia [49].

Future studies are needed to explore the potential of immersive VR to reduce the need for perioperative pharmacologic sedation [13]. For example, in one clinical study, VR distraction significantly reduced the dose of intraoperative propofol sedation during hand surgery, with no decline in any of the following measures: pain ratings, overall satisfaction, perioperative opioid dose, or post-operative functional outcome [50].

Limitations: Although promising, the current results must be interpreted with caution, given that case studies are inconclusive by their very nature [51]. Although validated, reliable standardized anxiety measures were used, the case study design we used limits the general applicability or generalizability of the results. Furthermore, the current study used a “within subject” design that has statistical advantages for small samples but is prone to the carryover effects of VR [52]. Carryover effects refer to a potential confound where a previous treatment (e.g., the no VR condition) can alter behavior (or ratings) in a subsequent experimental treatment (e.g., the VR condition). Carryover effects can limit the internal validity of a study and should be minimized [53]. The current study used a “washout” period between treatment conditions to help reduce carryover effects. Furthermore, in the current study, fortunately, the patient was randomized to receive no VR first (since no VR involved no treatment). Another concern is that the small sample size limits the generalizability of the results, a known limitation of all case studies. Carefully controlled studies with larger samples sizes are needed. Subjective self-reports are currently the gold standard for measuring anxiety. These measures indicate patients’ perceptions of how anxious or fearful they are. Future studies using long-term follow-up measures are needed. Individual differences in VR tolerance and preference could significantly affect VR’s applicability to a broader patient population. Further research with rigorous methodology and a more diverse participant pool is necessary to establish the credibility and practicality of using VR as a preoperative anxiety-reduction tool.

## 5. Conclusions

VR can help healthcare providers reduce fear and anxiety and increase fun during pre-op preparation. Immersive virtual reality may serve as a neuroprotective technique that can be used to reduce or avoid some of the neurotoxicity from pharmacologic sedation/anesthesia (e.g., helping to avoid oversedation). VR technology has recently become affordable and more user friendly, increasing the potential for widespread dissemination into medical practice. Additional research and development exploring the use of VR and/or mixed reality [36,54,55] before and potentially during and/or after surgery (in some cases) is recommended, using larger sample sizes, between-group designs, and randomization and blinding patients to different treatment conditions. 

## Data Availability

The original results presented in the study are included in the article/Supplementary Materials; further questions can be directed to the corresponding author.

## Supplementary Materials

The following supporting information can be downloaded at: https://www.mdpi.com/article/10.3390/healthcare11192697/s1, Figure S1: Visual analog scale.
