# Supplementary figures and images for: Using Immersive Virtual Reality Distraction to Reduce Fear and Anxiety before Surgery
Source: Healthcare (Basel). 2023 Oct 9;11(19):2697. doi: 10.3390/healthcare11192697 (PMC10572694; doi:10.3390/healthcare11192697)

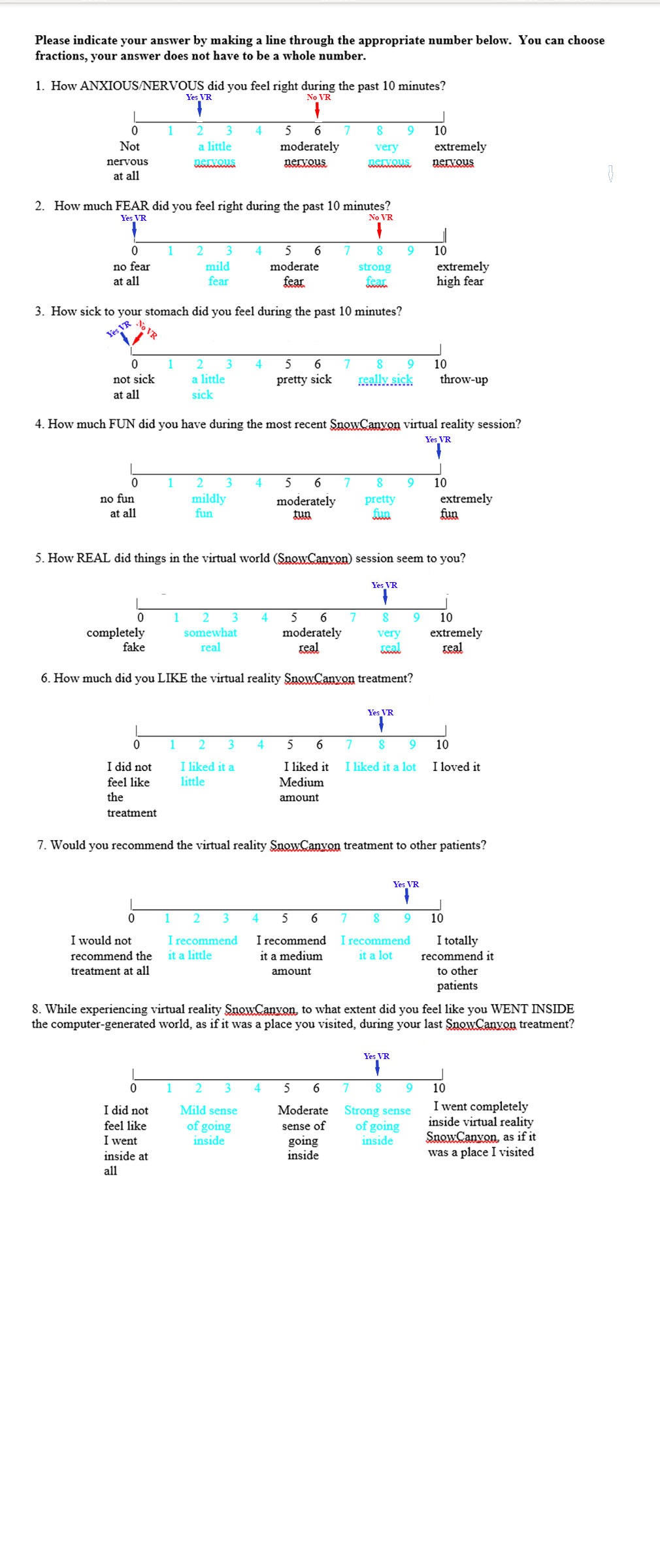

Supplement: Supplementary file 1 [file healthcare-11-02697-s001.zip › healthcare-2589366-supplementary.jpg]
